# Nipple Aspirate Fluid at a Glance

**DOI:** 10.3390/cancers14010159

**Published:** 2021-12-29

**Authors:** Susana I. S. Patuleia, Karijn P. M. Suijkerbuijk, Elsken van der Wall, Paul J. van Diest, Cathy B. Moelans

**Affiliations:** 1 Department of Pathology, University Medical Center Utrecht, Utrecht University, 3508 GA Utrecht, The Netherlands; s.i.schoutenpatuleia-7@umcutrecht.nl (S.I.S.P.); p.j.vandiest@umcutrecht.nl (P.J.v.D.); 2 Department of Medical Oncology, University Medical Center Utrecht, Utrecht University, 3508 GA Utrecht, The Netherlands; K.Suijkerbuijk@umcutrecht.nl (K.P.M.S.); e.vanderwall@umcutrecht.nl (E.v.d.W.)

**Keywords:** liquid biopsy, nipple aspirate fluid, biomarkers, microRNAs, breast cancer

## Abstract

**Simple Summary:**

Nipple aspirate fluid (NAF) is a promising source of markers for detection of breast cancer. NAF can be acquired via the nipple by aspiration using a suction device, which is well tolerated by women. Future possible applications of biomarkers for breast cancer derived from NAF could be (1) as a detection tool to identify the initiation of the cancer development process, (2) as an additional tool next to imaging (mammography and breast magnetic resonance imaging) or (3) as a replacement tool for when imaging is not advisable for women, such as during pregnancy and breastfeeding. With this paper, we present a narrative review and perspectives of NAF research at a glance.

**Abstract:**

Nipple aspirate fluid (NAF) is an intraductal mammary fluid that, because of its close proximity to and origin from the tissue from which breast cancer originates, is a promising source of biomarkers for early breast cancer detection. NAF can be non-invasively acquired via the nipple by aspiration using a suction device; using oxytocin nasal spray helps increase yield and tolerability. The aspiration procedure is generally experienced as more tolerable than the currently used breast imaging techniques mammography and breast magnetic resonance imaging. Future applications of NAF-derived biomarkers include their use as a tool in the detection of breast carcinogenesis at its earliest stage (before a tumor mass can be seen by imaging), or as a supporting diagnostic tool for imaging, such as when imaging is less reliable (to rule out false positives from imaging) or when imaging is not advisable (such as during pregnancy and breastfeeding). Ongoing clinical studies using NAF samples will likely shed light on NAF’s content and clinical potential. Here, we present a narrative review and perspectives of NAF research at a glance.

## 1. What Is Nipple Aspirate Fluid?

**Figure 1:**
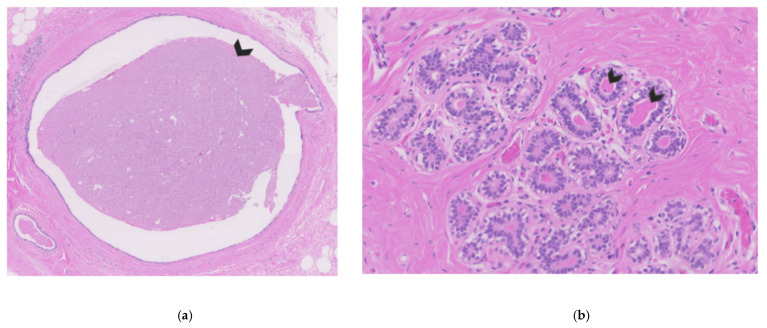
Breast tissue of a non-lactating breast. (**a**) Cross-section of breast ducts filled with nipple fluid on the inside (see arrow). (**b**) Cross-section of breast lobules with acini filled with eosinophilic nipple fluid on the inside (arrows indicate two examples).

NAF can be defined as the physiological fluid that is present in the breast ductal system (Figure 1). As indicated by the name, NAF can be acquired via the nipple by aspiration using a suction device, and, as such, non-invasively. This collection procedure differs from ductal lavage, a more invasive procedure in which a microcatheter is used to cannulate and flush the ducts [1]. Of relevance too is the difference in definition between NAF and pathological nipple discharge (PND). PND is defined as spontaneous, unilateral nipple discharge that can be an indication of benign (and rarely malignant) breast disease [2,3]. In contrast, NAF, which is fluid physiologically present in the breast ducts, in normal circumstances does not spontaneously leave the breast.

## 2. How Is NAF Produced?

**Figure 2:**
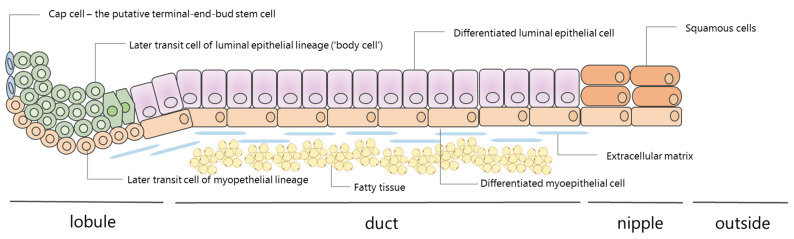
The mammary lobular–ductal system: cellular composition of the lobule, duct, and nipple (half linear representation). The breast contains around 5–12 mammary lobulo–ductal systems that start at the terminal end-bud and reach the nipple. Several lobulo–ductal structures can come together, leading to branching of this system into common ducts that end in the nipple. The mammary duct is composed of an outer layer of myoepithelial cells and an inner layer of luminal epithelial cells. At the end, near the nipple, there is a transition to squamous cells. Nipple aspirate fluid is probably produced by the inner luminal layer. The cap cells can take on either a myoepithelial lineage or a luminal epithelial lineage and therefore are thought to be multipotent stem cells. This scheme is adapted from Smalley and Ashworth [29] and Jakub et al. [30].

The backbone of the breast is the lobular–ductal system which branches into the breast from the nipple as has elegantly been shown in studies using contrast-injection in the nipple followed by mammography (galactography) [4] and in three-dimensional computer reconstructions [5,6]. The lobular–ductal system contains two main cell types, the luminal cells and the myoepithelial (basal) cells (Figure 2), and is surrounded by fatty tissue and extracellular matrix [7,8].

NAF is believed to be produced by the luminal epithelial cells (Figure 2), which are also responsible for breast milk production during lactation [9]. More specifically, the components present in NAF are thought to be synthesized within the breast epithelium and directly released into the ducts, and/or to be transferred from the circulation into the breast fluid [10]. The first theory is based on studies that have shown that components like estrogen, which can be produced in breast epithelial cells and stromal breast cells, have a higher concentration in NAF compared to serum [11]. The latter theory rests on studies that have found exogenously derived substances in ductal fluid, like nicotine in NAF of smokers [12,13,14,15]. The physiology of NAF also includes a reabsorption mechanism to blood or lymphatics. This has been shown in studies in which intraductal injections of India ink moved through the ductal walls to the lymphatics [14] and also by the fact that intraductal injections of cisplatin in mice led to systemic side effects [16].

NAF is in theory present in every breast. The success percentages of obtaining NAF are very high (77–95%) [17,18,19,20]. Unsuccessful aspiration can be explained by factors like the collection procedure, the number and duration of attempts, use of oxytocin to release NAF and whether the existing NAF is able to reach the nipple. Next to the procedure itself, it is believed that characteristics from women, like younger age [21,22], history of breastfeeding [21,23], not taking oral contraception [24], and soy consumption [25] could be good predictors for successful NAF collection; however, other studies do not confirm these data [23,26,27,28]. The presence of nipple fluid can microscopically be seen in breast tissues, as shown in Figure 1.

## 3. How Is NAF Collected?

**Figure 3:**
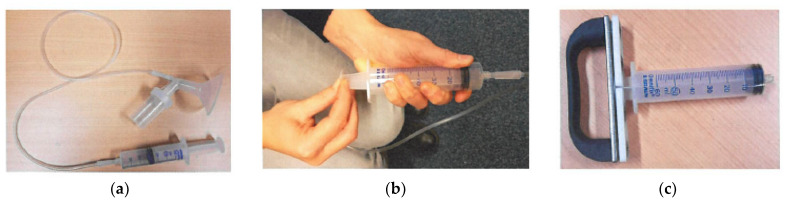
Pictures of materials used for manual nipple fluid aspiration procedure. (**a**) Interconnection with a plastic tube between the cup that is placed on the breast around the nipple and a syringe; (**b**) figure of the research nurse showing how to grasp the plunger of the syringe to create vacuum. (**c**) Ergonomic handle that we developed to attach to the plunger of the syringe.

NAF can be obtained by nipple fluid aspiration (NFA) with a (manual or electrical) suction device that creates negative pressure around the nipple. The manual suction device is comparable to a breast pump and consists of a cup that is placed on the breast around the nipple, a plastic tube, and a syringe, which are connected (Figure 3). The negative pressure is created manually by drawing the plunger of the syringe (that is at the one end of the plastic tube) back and forth. In our experience, this movement can be facilitated by the use of a handle that fits the plunger of the syringe, which makes this more ergonomic (see Figure 3c). The aspiration cup used can be a Sartorius cup or a commercially available breast pump (e.g., FirstCyte aspirator; Cytyc Corporation, Marlborough, MA [24,31,32]).

NFA is a non-invasive procedure that can be performed repeatedly and that allows obtaining a liquid biopsy from both breasts. Common steps of the procedure include the use of a scrubbing gel followed by cleansing the nipple surface with alcohol to remove keratin plugs clogging the orifices of the nipple ducts. Several pre-procedural and per-procedural additions have been proposed to improve the NFA success rate, such the application of warm compresses or heat pads to the breast [28], breast massage before and/or during the procedure [33,34,35], or repeated suction attempts [36,37,38]. Other approaches such as the application of an anaesthetic cream on the nipple and the intranasal administration of oxytocin have been suggested to diminish vacuum-induced pain and increase NAF collection success rate, respectively [17,38,39,40]. NFA requires a short training and can be mastered after 2 to 5 try-outs. Most studies collect NAF in the outpatient clinic setting, with the patient sitting in the upright position, but there are also a few studies that describe the collection of NAF from breast cancer patients directly prior to surgery under general anaesthesia [41,42].

## 4. Discomfort Associated with NFA

Tolerability of the NFA procedure has only been reported by a few studies. On a scale from 0 to 10, the discomfort of NFA has been rated with a mean score of 0.6 to 2.4 [17,26,39,40] in healthy female volunteers and women at high risk for breast cancer (Table 1). These scores are comparable to the scores given to a pap smear for cervical cancer screening and are generally lower than mammography, breast magnetic resonance imaging (MRI), physical breast examination and breastfeeding (see Table 1 for reported means). It is very encouraging to see that healthy women and women at high risk for breast cancer experience low discomfort with NFA, given that these are the women in whom a NAF test would especially be applicable.

## 5. Why Investigate NAF?

It is assumed that biomarkers in body fluids that are closer to the tumor or its target organ have more potential as a disease biomarker because they represent local pathological processes better than the more distant fluids such as serum and plasma that also receive cellular debris and other components from other body parts. Many biomarker classes have been investigated in NAF, such as hormones (e.g., estrogen, testosterone), tumor markers (e.g., carcinoembryonic antigen and prostate-specific antigen (PSA)) and biochemical components (e.g., aluminum). Moreover, NAF contains molecular markers, such as DNA [43,44], RNA, microRNA [45] and proteins [46,47]. These findings have been summarized in two reviews from Edward Sauter and Ferdinando Mannello et al. [46,47]. In Table 2, we provide an overview of the molecules and molecular changes found in NAF and the corresponding techniques used. Regarding cellular composition, NAF samples are known to be quite acellular or paucicellular, with reports of the presence of foam, white, red blood and/or (atypical) epithelial cells [46,48,49,50]. Given that breast tumors develop from ductal and/or lobular epithelium, investigating NAF as biofluid secreted by breast cells provides the opportunity to assess biomarkers directly originating from the breast. Moreover, the NAF procedure provides an intra-patient control to compare an affected breast with the other healthy breast of the same woman. As such, NAF is a potentially very valuable source of biomarkers that can serve as a diagnostic tool for breast disease, including breast cancer.

## 6. How Could NAF Be of Added Value as an Early Breast Cancer Detection Tool?

With current image-based breast cancer screening tools, breast cancer can only be detected once it has formed a mass. Liquid biopsy approaches such as NAF could be of added value by detecting breast cancer at an earlier or even pre-invasive stage.

Three recent studies have suggested how NAF (or other liquid biopsies) could be integrated in an early detection pathway for breast cancer [1,70,71]. Shaheed et al. suggests using NAF collection as a first step in screening for breast cancer, followed by sample analysis by mass spectroscopy [1]. Another suggestion by Shaheed et al. is the use of an easy-to-interpret NAF self-collection kit [1]. These elements are also included in the recent publication of Jiwa et al., that describes a possible workflow for management of breast screening that integrates NAF [71]. A more specific potential application of NAF could be, as Zubor et al. suggests, the use of liquid biopsies as an adjunct to supplemental MRI for women with dense breasts or (additional) breast cancer related risk factors [70].

**Figure 4:**
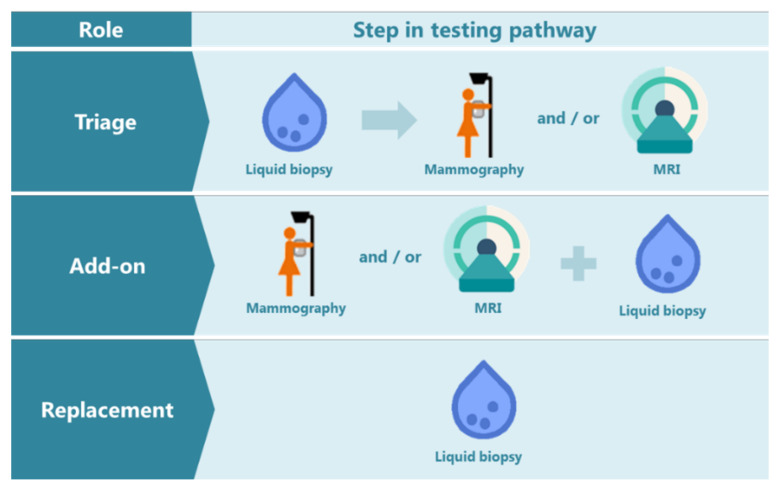
The possible roles of a liquid biopsy test in the breast cancer detection pathway: triage, add-on, or replacement test. MRI: Magnetic resonance imaging. The mammography schematic icon is adapted from the Dutch information folder for participation in the National Programme for Breast Cancer Screening [73].

These proposed roles of NAF or other liquid biopsies for early detection of breast cancer, together with our views, are summarized as shown in Figure 4. In general, there are three possible roles for a new biomarker test in an already existing detection pathway, namely as a triage, add-on or replacement test [72]. A triage test is used before the existing imaging method; only women with an abnormal biomarker test result would continue in the detection pathway. Such a triage test may be less accurate but should provide advantages such as simplicity and low costs. An add-on test could be positioned after or together with imaging to identify false-positives or false-negatives and hence, avoid unnecessary breast biopsies and missing tumors, respectively. The third role, namely a replacement test, could be of value if there are actionable consequences to halt further tumor development or when imaging as a screening or diagnostic tool is not advisable, such as during pregnancy and breastfeeding. In the end, the true application of a NAF test will be dependent on the accuracy of the biomarkers for disease detection. In addition, it will be dependent on the characteristics of the cohorts used in clinical studies to test the NAF samples. Last but not least, involvement of medical decision-making experts and patient advocate groups are of utmost importance to discuss the translational applications.

## 7. Challenges in Using NAF as a Biomarker Source

Even though NAF holds promise as a biomarker source for detection of breast cancer, some challenges emerge when handling NAF. An important limitation is the small volume (ranging from 1 to 500 µL [74]). Furthermore, viscosity and different colors have been shown to affect biomarker analyses [10,75]. In our experience, volume and viscosity are an issue when using fluorescence-activated cell sorting, spectrophotometry and microfluidics. Ways to render viscous, heterogeneous NAF specimens to a homogenous state, could be the use of mechanical liquefaction, such as vortexing with the aid of glass beads or sonication and/or chemical liquefaction with the use of mucolytic buffers and centrifugation through a QIAshredder homogenizer prior to nucleic acid extraction [76]. Since pipetting is also hampered by viscous samples, a requirement would be to have automatic positive displacement pipettes to ensure accuracy and reproducibility. Still, current relevant biomarker techniques like real time quantitative reverse transcription PCR (RT-qPCR) [45,55] and mass spectrometry [15,47] have been shown to be feasible for NAF samples.

Another limitation of NAF is that it is unclear from which of the 5–12 ducts within the breast the NAF droplets originates, which comes with the risk of not acquiring NAF from the duct where carcinogenesis takes place. Moreover, it could be that larger tumors may occlude the ducts distally and hence, obstruct NAF flow for collection; from our experience, NFA success percentages from the affected and unaffected breast in women with breast cancer are comparable (data not shown). Still, if NAF is to be used in the context of early breast cancer detection by making use of its biomarkers to signal carcinogenesis before an obstructing tumor mass has been formed, this should not be a matter of concern.

## 8. Historical Overview of Studies Focusing on NAF

NAF was first described by Papanicolaou et al. [77] and Fleming [78] in the mid-1950s. In 1977, Sartorius et al. developed a “Sartorius” suction cup for NFA which consisted of a small plastic cup attached to a 10-mL syringe by a short plastic tube [79]. Sartorius and his colleagues pioneered the epidemiological research using NAF samples by building up a cohort of over 3000 women of whom they attempted NFA [24]. Data from these cohorts was used for several publications, especially by himself, Eileen King, Nicholas Petrakis and Margaret Wrensch. So far, over 200 publications about NAF have been written. In order to give a historical overview of research related to NAF, we shortly describe the work of selected researchers that have especially dedicated themselves to NAF, namely Otto Sartorius, Nicholas Petrakis, Ferdinando Mannello, Edward Sauter, and Paul van Diest and Elsken van der Wall. The search strategy that led to this selection comprised a PubMed search for all articles that included the terms “nipple aspirate fluid” or “nipple aspiration”, together with snowballing. This was followed by a selection of articles by authors that had published more than four articles as first or last author, not including reviews. When co-authorship occurred often, selection was performed for the most senior author. A timeline with their list of publications is represented in Figures S1–S4.

The work of Petrakis et al. (Figure S1) about NAF was first published in 1975 and comprised investigations on success percentage of NFA, cytology analysis of NAF, and the association of both with breast cancer risk factors. The initial work was mainly focused on the success percentages of acquired NAF and relation to age, ethnicity, menopausal status, and cerumen type. A higher success rate of NAF yield was seen in Caucasians, women younger than 50 years old and with wet type cerumen [80,81], which was later confirmed in another study [82]. Additional variables with a positive influence on success rate of NFA were a higher fat consumption [83], history of parity and of breastfeeding [84]. Another scope of research was the relation between cells found in NAF and the risk of developing breast cancer [85,86]. In one publication it was shown that, when adding cellular atypia found in NAF, a minimal improvement of the Gail model for risk prediction was obtained [87]. In another publication, they described that atypical cells could be found in healthy women, women with benign breast disease and women with breast cancer, with increasing percentages per group (15%, 32%, and 64%, respectively) [88]. Later, for the first time, the association was made between cellular atypia in NAF and high mammographic density (>50% density) [50]. Moreover, foam cells were for the first time characterized in NAF [89]. An interventional study investigating the effect of the intake of soy protein showed that this led to an increased NAF secretion and the appearance of hyperplastic epithelial cells in NAF of premenopausal women [25]. Lastly, they analyzed the biochemical content of NAF for cholesterol (as it could be a potential carcinogen) [90] and lactose (as it could be considered a measure of secretory activity) [91]. In 1993, Petrakis gave a distinguished award lecture summarizing his work on NAF until then [92].

The work of Mannello et al. (Figure S2) started with publications in the beginning of the 21st century and mostly focused on the biochemical constituents of nipple aspirate such as P-cadherin [93], proteins involved in the of lipid peroxidation and oxidative stress [94,95,96], C-reactive protein [97], and aluminum [98,99,100]. Methodologically, for these investigations, Mannello et al. usually compared NAF from cohorts of healthy women with cohorts of women with breast cancer; comparing biomarker concentrations between NAF, plasma, and serum. Most of his studies were observational, but he also performed an interventional study by investigating the levels of urokinase-type plasminogen activator (uPa) in NAF after oral administration of colecoxib, a non-steroidal anti-inflammatory drug [101]. He also wrote four reviews summarizing current knowledge about proteins, hormones and aluminum detected in NAF of breast cancer patients compared to healthy controls [47,102,103,104]. His latest publication was a comment reflecting on the potential of investigating biomarkers in NAF [105].

Another researcher with NAF as a clear line of investigation is Sauter, who has compiled an impressive number of publications on NAF over almost 30 years (Figure S3). Sauter focused on the levels of markers in NAF, especially prostate-specific antigen (PSA) (PSA is produced by breast tissue) [67,106,107], and PSA’s association with hormones such as progesterone [108] and testosterone [64] and insulin-like growth factor binding protein-3 [109]. Specifically, PSA levels in NAF were inversely associated with the presence of breast cancer, suggesting a possible role for PSA to help establish breast cancer risk [67]. Moreover, the levels of PSA were investigated in breast cancer patients, and shown to decrease with advanced breast cancer stages, and to be able to better predict disease in pre-menopausal women compared to post-menopausal women [69]. Next to his focus on the biochemical composition of NAF [44,56,59,60,62,93,95,96,107,110,111,112,113,114,115,116], he also investigated the cellular components of NAF. In one study he established that there was no cellular variation in NAF along the menstrual cycle of 15 healthy women by evaluating weekly acquired NAF samples [117]. He also performed interventional studies where it was investigated whether celecoxib had effect on prostaglandin E2 concentrations in NAF from women at increased risk of breast cancer [118]. His latest publication is an editorial about a publication of our group [119].

The work of our group (Figure S4) on NAF started in 2007 with publications focused on the use of oxytocin nasal spray prior to the NFA procedure, which proved to yield a higher success rate, to be safe and to be well accepted by the participating women [17,39,40]. This was followed by a publication on methylation levels of tumor suppressor genes in NAF samples from women with breast cancer compared to healthy women. This study showed that cancerous nipple fluid contained increased levels of methylation biomarkers compared to healthy nipple fluid, albeit with an area under the curve that was lower than current imaging methods [120,121]. We further focused on NAF microRNA analysis, based on the fact that these biomarkers can be detected in several liquid biopsies and can make up a signature for oncological disease. We have proven that microRNAs (miRNAs)are measurable in NAF by RT-qPCR [45] and are currently investigating the feasibility of small RNA sequencing for miRNA detection in NAF samples. Now that our group has piled up a large sample biobank with blood and NAF from healthy women, women with breast cancer and serial samples from women at increased risk of developing breast cancer, more publications will soon follow [122,123,124]. These data will reveal whether early detection before mammography can be reliably achieved in NAF by means of miRNAs. The practical aspects of building up these cohorts have been described in a recent publication [125], followed by a publication reporting the technical aspects of analyzing biomarkers in NAF samples of different appearance [75]. We are currently investigating the role of miRNAs in the development of high breast mammographic density and the consequently increased risk of breast cancer, by using NAF as a surrogate for the breast microenvironment.

The latter three publications comprise the most recent publications about NAF, together with publications from George et al. [126] and Jiwa et al. [48,71,127]. The first described a proteomic evaluation of NAF [126] whereas the latter described in a systematic review that the diagnostic accuracy of nipple smear cytology is limited by poor sensitivity [48,127] and, in a questionnaire-based study, that there is a great readiness of women to undergo NFA [71]. Based on ongoing clinical studies using NAF samples (see Table 3), future data on NAF’s content and clinical potential will be generated and reported. The state of the art of NAF research is summarized in Box 1 and a list of past reviews about NAF is shown in Table 4.

Box 1 **Summary of the state of the art of NAF research: what we know, advantages, hurdles to be aware of, and recommendations and needs in NAF studies.**

**Box 1|Summary**

**What we know**

- Nipple aspirate fluid (NAF) is a physiological fluid that is produced by the luminal layer of the breast lobules and ducts
- Nipple fluid aspiration (NFA) is well tolerated by women
- NAF can be acquired in the majority of women
- Many biomarkers can be found in NAF, such as DNA, RNA, microRNA and proteins
- Cytology assessment in NAF has low diagnostic accuracy

**Advantages**

- NAF originates from the location where breast cancer arises
- NAF can be acquired repeatedly, easily and non-invasively
- Bilateral NAF samples allow intra-patient control analyses

**Hurdles to be aware of**

- NAF samples are of low volume, can be viscous, have low cellularity and have different colors, which may affect biomarker analysis
- It is unclear which duct NAF derives from

**Recommendations and needs in NAF studies**

- For the NFA procedure, use oxytocin nose spray to increase success rate and tolerability for the woman, and use an ergonomic handle for the research nurse performing the procedure
- Combine nipple fluid research with imaging results and include anthropomorphic measures and risk factors for breast cancer
- Development of technologies that are feasible for detection and interpretation of biomarkers in samples that are viscous and of low volume
- To reduce sample viscosity, use mechanical and/or chemical liquefaction in sample processing steps
- Report NAF biomarker results in diagnostic accuracy values in order to be able to interpret their translational role
- Involve medical decision making experts and patient advocate groups to discuss the potential use of liquid biopsies in early detection
- Develop a self-test for NFA collection and interpretation

## 9. Conclusions

NAF is a physiological fluid that rests in the ductal tree of the breast and can easily be obtained by non-invasive aspiration. It is an established source of biomarkers that deserves to be investigated for its potential application in breast cancer management such as in screening or as a confirmatory additive tool in imaging diagnostics. As research on the role of NAF is still actively being performed by many research groups, including our own, more data about NAF and its potential clinical value is expected to be reported in the near future.

## Figures and Tables

**Table 1:** Reported mean discomfort rates (on a scale from 0–10) for NFA (nipple fluid aspiration) compared to pap smear, mammography, breast magnetic resonance imaging (MRI), breastfeeding and breast examination. Abbreviations: N.R., not reported; H, healthy female volunteers; HR: women at high risk of developing breast cancer.

First Author (Year) (Reference)	NFA	Pap Smear	Mammography	MRI	Breast-Feeding	Breast Examination	Cohort	Number of Women
Klein et al. (2001) [26]	2.4	2.2	4.6	N.R.	6.8	N.R.	H	25
Suijkerbuijk et al. (2007) [39]	1.3	N.R.	4.3	N.R.	1.9	N.R.	H	67
Suijkerbuijk et al. (2010) [17]	0.6	N.R.	4.9	2.6	1.8	1.1	HR	90
de Groot et al. (2015) [40]	0.7	N.R.	5.2	3.55	2.5	1.15	HR	451

**Table 2:** Summary of molecules or molecular changes found in NAF and used techniques, their advantages, and limitations. This information was retrieved from the reviews of Sauter [46] and Mannello et al. [47]. Abbreviations: N.A.: not applicable.

Molecules or Molecular Changes Detected in NAF		Technique		References
	Technique Used in NAF	Advantage(s)	Limitation(s)	
DNA mutagens	Ames test	Simple to perform Inexpensive	Relatively long time to perform analysis	[26,51,52]
Methylation changes in DNA	Methylation-specific PCR (MSP) technique	High sensitivity and reproducibility of quantitative measurements High resolution of target regions High-throughput capability	False-positive results Variability of results due to assay conditions	[44]
Mutations in mitochondrial DNA	Polymerase chain reaction (PCR)	Accurate quantitation High molecular sensitivity Ease of use	Low/medium throughput	[53]
Microsatellite markers in DNA to investigate loss of heterozygosity or microsatellite instability alterations				[43,53]
Mutations in the mitochondrial genome (mtgenome)	Sequencing the entire mitochondrial genome and mitochondrial resequencing array 2.0 (MCv2)	High sensitivity Automation, which leads to cost benefits in reagents, labor, time-to-results, ease, and accuracy of data interpretation	Data interpretation	[54]
RNA and microRNA	Real time quantitative reverse transcription PCR (RT-qPCR)	High sensitivity and specificity High dynamic range Suitable for quantification	Low/medium throughput	[45,55]
Proteome	Mass spectrometry Surface-enhanced laser desorption/ionization (SELDI)-TOF technique and Matrix-Assisted Laser Desorption Ionization (MALDI)-TOF mass spectrometry	Extremely sensitive (feasible with very small sample quantities) Able to identify unknown components in liquid samples Very precise, rapid, and sensitive Rapid turnaround time	Costly Requires a skilled technician Generated data require expertise for interpretation	[56,57,58,59]
	2D polyacrylamide gel electrophoresis (PAGE)	Allows accurate analysis of thousands of proteins in a single run	Many sample-handling steps Limited reproducibility Not automated for high throughput analysis	[60]
	Liquid chromatography	Extremely quick and efficient	Is subject to greater peak or band-broadening and therefore to lower resolution	[61]
	Enzyme-linked immunosorbent assay (ELISA)	Simple procedure High specificity and sensitivity Easily automated	Is subject to high background which affects the sensitivity of the assay	[60]
Biochemical substances (e.g., α-lactalbumin, immunoglobulins, lipids, fatty acids, proteins, cholesterol, and cholesterol oxidation products)	Gas liquid chromatography (GLC, for lipids)	High efficiency: GLC allows the separation of sample components in a reasonable time	Only for thermally stable and volatile compounds One time use per sample as it leads to sample destruction	[14]
	Fluorescence technique of Tappel (for lipid peroxidation)	N.A. (not used anymore)	N.A. (not used anymore)	[14]
	Immunoelectrophoresis (for alpha-lactalbumin) and rocket immunoelectrophoresis (RIE, for immunoglobulins)	Good stability of the reagents Performance similar to ELISA	Time-consuming	[14]
Hormones (e.g., estrogens, androgens, progesterone, dehydroepiandrosterone Sulfate (DHEAS), prolactin, growth hormone and leptin, and the growth factors epidermal growth factor, transforming growth factor-α, vascular endothelial growth factor (VEGF) and basic fibroblast growth factor (bFGF))	Immunoassays (such as enzyme-linked immunosorbent assay (ELISA))	High efficiency	Antibody instability Insufficient blocking of immobilized antigen can lead to false results	[62,63,64,65,66]
Tumor antigens (e.g., carcinoembryonic antigen (CEA) and prostate-specific antigen (PSA))	Immunoassays (fluorescence immunoassay (FIA) for PSA, immunoenzymometric assay for CEA)	Highly sensitive and highly specific High efficiency	Interference of filtration step in final emission of FIA	[64,67,68,69]

**Table 3:** List of registered trials on nipple aspiration fluid identified by searches in https://clinicaltrials.gov/, accessed on 25 December 2021. Trialregister.nl, ISRCTN and PubMed. The following syntax was used for a search on Pubmed: (“protocol”[Title]) AND (nipple aspirate fluid OR nipple aspirate*)).

Title of the Clinical Study	Characteristics of Participants	Aims Related to Nipple Aspirate Fluid Samples	Status	Source	Reference
Nipple Aspirate Fluid in Detecting Breast Cancer	Participants are healthy volunteers >40 years who undergo collection of nipple aspirate fluid from both breasts.	Nipple aspiration fluid samples will be compared between breast cancer participants and healthy participants. will perform the logistic regression model for each biomarker that shows any difference between the breast cancer patients and healthy individuals. Then we will include multiple biomarkers in one model while controlling for confounders.	Recruiting	Clinicaltrials.gov (accessed on 25 December 2021)	[128]
Early detection of Hereditary Breast Cancer by Monitoring MicroRNA expression in Nipple Aspirate Fluid	Women at high risk of developing breast cancer	- To establish biomarker profiles in NAF, follow them in time and establish a correlation with breast cancer development. - To determine threshold values of these biomarkers that point to a significant risk of imminent breast cancer development thereby indicating the right time of prophylactic breast surgery. (Trial NL8661)	Recruitment stopped in 2021. Lab analyses ongoing.	trialregister.nl (accessed on 25 December 2021)	[122]
Breast Cancer Biomarkers in Nipple Aspirate Fluid and Blood in Healthy Women	Participants are healthy volunteers ≥45 years	The main study parameters is the degree and patterns of microRNA expression in NAF and blood of healthy women, and to compare this with the pattern of women with breast cancer (ORNAMENT study, Trial NL6031)	Recruitment stopped in 2021. Lab analyses ongoing.	trialregister.nl (accessed on 25 December 2021)	[123]
The ORNAMENT study: A multicenter, crOss sectional, study to assess microRNA expression in Nipple Aspirated Fluid, blood and tuMor material in women with primary brEast caNcer compared with healthy conTrols.	Women with pathologically established non-metastasized invasive breast carcinoma	To assess the microRNA expression levels in nipple aspiration fluid obtained just before primary surgery. These will be compared to the microRNA expression levels in NAF obtained from healthy controls (Trial NL8987).	Recruitment stopped in 2021. Lab analyses ongoing.	trialregister.nl (accessed on 25 December 2021)	[124]
Physical activity and dietary counseling and supervised group exercise for first-time pregnant women—a feasibility study of a controlled trial	Women who gave birth and stopped breastfeeding	Secondary outcome: Levels of selected breast cancer risk markers (hormones, growth factors) in blood and nipple aspirate fluid (only in postpartum women)	Active, but not recruiting	ISRCTN registry	[129]
Phase II study of metformin for reduction of obesity-associated breast cancer risk: a randomized controlled trial protocol	Eligible participants will be randomized to receive metformin 850 mg BID (*n* = 75) or placebo (*n* = 75) for 12 months.	Exploratory outcomes: changes in metabolomic profiles in plasma and nipple aspirate fluid.	Not recruiting	PubMed	[130]

**Table 4:** List of reviews about nipple aspirate fluid or where nipple aspirate fluid is mentioned amongst other approaches in the manuscript. Abbreviations: R, review; SR, systematic review; W, workshop.

Type	Title	Year	Author(s)	References
R/W	Physiologic, biochemical, and cytologic aspects of nipple aspirate fluid.	1986	Petrakis	[14]
SR	Factors associated with obtaining nipple aspirate fluid: analysis of 1428 women and literature review.	1990	Wrensch et al.	[21]
R	Epidemiology and prevention of breast cancer.	1996	Kelsey and Bernstein	[131]
R	Nipple aspirate fluid in relation to breast cancer.	1999	Phillips et al.	[132]
R	Lavage and nipple aspiration of breast ductal fluids: a source of biomarkers for environmental mutagenesis	2002	Klein and Lawrence	[133]
R	Breast cancer chemoprevention: current challenges and a look toward the future.	2002	Fabian and Kimler	[134]
R	Ductal lavage, nipple aspiration, and ductoscopy for breast cancer diagnosis	2003	Dooley WC	[135]
R	The role of ductal lavage in the management of women at high risk for breast carcinoma	2004	Khan SA	[136]
R	Ductal lavage in the screening of high-risk women	2004	Kenney PJ, Ellison MC	[137]
R	The local hormonal environment and related biomarkers in the normal breast	2005	Khan SA et al.	[138]
R	The Fourth International Symposium on the Intraductal Approach to Breast Cancer, Santa Barbara, California, 10–13 March 2005	2005	King BL, Love SM et al.	[139]
R	Improved peak detection and quantification of mass spectrometry data acquired from surface-enhanced laser desorption and ionization by denoising spectra with the undecimated discrete wavelet transform.	2005	Coombes et al.	[140]
R	Breast-tissue sampling for risk assessment and prevention.	2005	Fabian, C J et al.	[141]
R	The intraductal approach to breast cancer biomarker discovery	2006	Dua et al.	[32]
R	The clinical applications of mammary ductoscopy	2006	Escobar PF et al.	[142]
R	Human body fluid proteome analysis	2006	Hu, Shen et al.	[143]
R	Breast ductal secretions: clinical features, potential uses, and possible applications	2007	Lang JE, Kuerer HM.	[144]
R	Proteomics of nipple aspirate fluid, breast cyst fluid, milk, and colostrum.	2007	Ruhlen and Sauter	[145]
R	Proteomic analysis of breast tissue and nipple aspirate fluid for breast cancer detection.	2007	Ruhlen and Sauter	[146]
R	Proteomic approaches for serum biomarker discovery in cancer.	2007	Maurya et al.	[147]
R	Human breast biomonitoring and environmental chemicals: use of breast tissues and fluids in breast cancer etiologic research.	2007	LaKind et al.	[148]
R	Molecular analysis of nipple fluid for breast cancer screening	2008	Suijkerbuijk et al.	[149]
R	Analysis of the intraductal microenvironment for the early diagnosis of breast cancer: identification of biomarkers in nipple-aspirate fluids.	2008	Mannello	[150]
R	Nutrients and nipple aspirate fluid composition: the breast microenvironment regulates protein expression and cancer aetiology.	2008	Mannello, et al.	[74]
R	Increased shedding of soluble fragments of P-cadherin in nipple aspirate fluids from women with breast cancer.	2008	Mannello et al.	[93]
R	Intracrinology of breast microenvironment: hormonal status in nipple aspirate fluid and its relationship to breast cancer.	2009	Mannello et al.	[104]
R	Protein profile analysis of the breast microenvironment to differentiate healthy women from breast cancer patients.	2009	Mannello et al.	[47]
R	Non-invasive proteomics-thinking about personalized breast cancer screening and treatment	2010	Debald M et al.	[151]
R	Breast cancer risk assessment, prevention, and the future.	2013	Green, Victoria L	[152]
R	Aluminium and breast cancer: Sources of exposure, tissue measurements and mechanisms of toxicological actions on breast biology.	2013	Darbre et al.	[102]
R	Resolving breast cancer heterogeneity by searching reliable protein cancer biomarkers in the breast fluid secretome.	2013	Mannello and Ligi	[103]
R	The human mammary gland as a target for isoflavones: how does the relation vary in individuals with different ethnicity?	2013	Maskarinec, Gertraud	[153]
R	The cancer secretome, current status and opportunities in the lung, breast and colorectal cancer context.	2013	Schaaij-Visser et al.	[154]
R	Development of a novel approach for breast cancer prediction and early detection using minimally invasive procedures and molecular analysis: how cytomorphology became a breast cancer risk predictor.	2015	Masood, Shahla	[155]
SR	Proliferative epithelial disease identified in nipple aspirate fluid and risk of developing breast cancer: a systematic review.	2015	Hornberger et al.	[156]
R	The In’s and Out’s of Ductography: A Comprehensive Review.	2016	Sheiman et al.	[4]
R	Evaluation of nipple aspirate fluid as a diagnostic tool for early detection of breast cancer.	2018	Shaheed et al.	[1]
R	The power of small changes: Comprehensive analyses of microbial dysbiosis in breast cancer.	2019	Parida, Sheetal and Sharma, Dipali	[157]
SR	Diagnostic Accuracy of Nipple Aspirate Fluid Cytology in Asymptomatic Patients: A Meta-analysis and Systematic Review of the Literature	2020	Jiwa N et al.	[48]
R	Lessons Learned from Setting Up a Prospective, Longitudinal, Multicenter Study with Women at High Risk for Breast Cancer	2020/2021	Patuleia SIS, Hagenaars SC et al.	[125]
R	Non-Invasive Biomarkers for Early Detection of Breast Cancer	2020	Li J et al.	[158]

## Supplementary Materials

The following are available online at https://www.mdpi.com/article/10.3390/cancers14010159/s1, Figures S1–S4. Timeline of published articles of Petrakis (S1), Mannello (S2), Sauter (S3), and Van Diest/Van der Wall (S4).
