# Supplementary material for: Nipple Aspirate Fluid at a Glance
Source: Cancers (Basel). 2021 Dec 29;14(1):159. doi: 10.3390/cancers14010159 (PMC8750428; doi:10.3390/cancers14010159)
Supplement: Supplementary file 1 [file cancers-14-00159-s001.zip › cancers-1509122-supplementary.pdf]

**Figure S1**

## Timeline of Petrakis

**1975** Association of race, age, menopausal status, and cerumen type with breast fluid secretion in nonlactating women, as determined by nipple aspiration.

### 1980s

**1981** Epidemiology of Breast Fluid Secretion: Association With Breast Cancer Risk Factors and Cerumen Type.
Genetic-environmental interactions in relation to low dose studies: a possible model from breast cancer.

**1982** Epithelial dysplasia in nipple aspirates of breast fluid: association with family history and other breast cancer risk factors.

**1983** Nipple aspirate cytology for the study of breast cancer precursors.

**1984** Analytic studies of foam cells from breast cancer precursors.

**1985** Nipple aspirates of breast fluid and the epidemiology of breast disease.

**1986** Physiologic, biochemical, and cytologic aspects of nipple aspirate fluid.

**1987** Breast fluid cholesterol and cholesterol epoxides: relationship to breast cancer risk factors and other characteristics.

**1989** Nipple aspirate fluids in adult nonlactating women--lactose content, cationic Na+,  K+, Na+/K+ ratio, and coloration.

### 1990s

**1990** **Cerumen phenotype and proliferative epithelium in breast fluids of U.S.-born vs.  immigrant Asian women: a possible genetic-environmental interaction.**

**Figure S1**

**Timeline of Petrakis**

**1992** Breast cancer incidence in women with abnormal cytology in nipple aspirates of breast fluid.
The association of dietary fat with ability to obtain breast fluid by nipple aspiration.

**1993** Nipple aspirate fluid in epidemiologic studies of breast disease.
ASPO Distinguished Achievement Award Lecture. Studies on the epidemiology and natural history of benign breast disease and breast cancer using nipple aspirate fluid.
Breast cancer risk associated with abnormal cytology in nipple aspirates of breast fluid and prior history of breast biopsy

**1994** Association of abnormal nipple aspirate cytology and mammographic pattern and density.

**1996** Stimulatory influence of soy protein isolate on breast secretion in pre- and postmenopausal women.

**1998** Birthplace and yield of nipple aspirate fluid in Chinese women.

2000s

**2001** Breast cancer risk in women with abnormal cytology in nipple aspirates of breast fluid.

**2004** Multiple sampling for increasing the diagnostic sensitivity of nipple aspirate fluid for atypical cytology.

**2005** Nipple aspirate fluid cytology and the Gail model for breast cancer risk assessment in a screening population.

**Figure S2**

## Timeline of Mannello

### 2000s

**1999** Molecular forms and ultrastructural localization of prostate-specific antigen in nipple aspirate fluids.

**2000** Biochemical and ultrastructural features of human milk and nipple aspirate fluids.

**2003** Zymographic analyses and measurement of matrix metalloproteinase-2 and -9 in nipple aspirate fluids.

**2007** Do nipple aspirate fluid epithelial cells and their morphology predict breast cancer development?
The 8-epimer of prostaglandin F(2alpha), a marker of lipid peroxidation and oxidative stress, is decreased in the nipple aspirate fluid of women with breast cancer.

**2008** Nipple aspirate fluids from women with breast cancer contain increased levels of group IIa secretory phospholipase A2.
Increased levels of erythropoietin in nipple aspirate fluid and in ductal cells from breast cancer patients.
Increased shedding of soluble fragments of P-cadherin in nipple aspirate fluids from women with breast cancer.
Nutrients and nipple aspirate fluid composition: the breast microenvironment regulates protein expression and cancer aetiology.
PA is upregulated by high dose celecoxib in women at increased risk of developing breast cancer.
Analysis of the intraductal microenvironment for the early diagnosis of breast cancer: identification of biomarkers in nipple-aspirate fluids.

**2009** Protein profile analysis of the breast microenvironment to differentiate healthy women from breast cancer patients.
Intracrinology of breast microenvironment: hormonal status in nipple aspirate fluid and its relationship to breast cancer.
Protein oxidation in breast microenvironment: Nipple aspirate fluid collected from breast cancer women contains increased protein carbonyl concentration.

### 2010s

**2010** Detection of superoxide dismutase-1 in nipple aspirate fluids: a reactive oxygen species-regulating enzyme in the breast cancer microenvironment.
Iron-binding proteins and C-reactive protein in Nipple Aspirate Fluids: role of Iron-driven inflammation in breast cancer microenvironment?

**2011** Aluminium and human breast diseases.
Analysis of aluminium content and iron homeostasis in nipple aspirate fluids from healthy women and breast cancer-affected patients.

**Figure S2**

# Timeline of Mannello

2020s

**2013** Resolving breast cancer heterogeneity by searching reliable protein cancer biomarkers in the breast fluid secretome.
Aluminium and breast cancer: Sources of exposure, tissue measurements and mechanisms of toxicological actions on breast biology.
Aluminium, carbonyls and cytokines in human nipple aspirate fluids: Possible relationship between inflammation, oxidative stress and breast cancer microenvironment.

**2017** New horizon for breast cancer biomarker discoveries: What might the liquid biopsy of nipple aspirate fluid hold?

**Figure S3**

# Timeline of Sauter

**1996** Prostate-specific antigen levels in nipple aspirate fluid correlate with breast cancer risk.

**1997** Nipple aspirate fluid: a promising non-invasive method to identify cellular markers of breast cancer risk.

**1998** Prostate-specific antigen production in the female breast: association with progesterone.

**1999** Biological markers of risk in nipple aspirate fluid are associated with residual cancer and tumour size.

## 2000s

**2000** Expression of a prostate-associated protein, human glandular kallikrein (hK2), in breast tumours and in normal breast secretions.

**2001**
Prostate-specific antigen levels in nipple aspirate fluid.
Nipple aspirate cytology and pathologic parameters predict residual cancer and nodal involvement after excisional breast biopsy.
Cellular characteristics of nipple aspiration fluid during the menstrual cycle in healthy premenopausal women.

**2002**
Circulating testosterone and prostate-specific antigen in nipple aspirate fluid and tissue are associated with breast cancer.
Proteomic analysis of nipple aspirate fluid to detect biologic markers of breast cancer.
Prostate-specific antigen and insulin-like growth factor binding protein-3 in nipple aspirate fluid are associated with breast cancer.
High basic fibroblast growth factor levels in nipple aspirate fluid are correlated with breast cancer.
Ethnic variation in kallikrein expression in nipple aspirate fluid.
Prostate-specific antigen in nipple aspiration fluid: menstrual cycle variability and correlation with serum prostate-specific antigen

**2003**
Microsatellite changes in nipple aspirate fluid and breast tissue from women with breast carcinoma or its precursors.
Association of uPA, PAT-1, and uPAR in nipple aspirate fluid (NAF) with breast cancer.
Nipple aspirate fluid expression of urokinase-type plasminogen activator, plasminogen activator inhibitor-1, and urokinase-type plasminogen activator receptor predicts breast cancer diagnosis and advanced disease.

**2004**
Association of kallikrein expression in nipple aspirate fluid with breast cancer risk.
Detection of breast cancer in nipple aspirate fluid by CpG island hypermethylation.
Prostate-specific antigen expression in nipple aspirate fluid is associated with advanced breast cancer.
Leptin expression in breast nipple aspirate fluid (NAF) and serum is influenced by body mass index (BMI) but not by the presence of breast cancer.
Lack of effect of celecoxib on prostaglandin E2 concentrations in nipple aspirate fluid from women at increased risk of breast cancer.
Proteomic analysis to identify breast cancer biomarkers in nipple aspirate fluid.

**2005**
Mitochondrial DNA mutations in breast cancer tissue and in matched nipple aspirate fluid.
Proteomic analysis of nipple aspirate fluid using SELDI-TOF-MS.
Thomsen-Friedenreich and Tn antigens in nipple fluid: carbohydrate biomarkers for breast cancer detection.
Analysis of nipple aspirate fluid for diagnosis of breast cancer: an alternative to invasive biopsy.

**2006**
Nipple aspirate fluid color is associated with breast cancer.
Celecoxib decreases prostaglandin E2 concentrations in nipple aspirate fluid from high risk postmenopausal women and women with breast cancer.

**Figure S3**

## Timeline of Sauter

**2010s**

**2007** Do nipple aspirate fluid epithelial cells and their morphology predict breast cancer development?
The 8-epimer of prostaglandin F(2alpha), a marker of lipid peroxidation and oxidative stress, is decreased in the nipple aspirate fluid of women with breast cancer.
Biologic markers of breast cancer in nipple aspirate fluid and nipple discharge are associated with clinical findings.
Prospective multicenter trial to determine the feasibility of collection and predictive ability of breast fluid analysis in postmenopausal women receiving SERMs.
Increased expression of the inflammatory protein YKL-40 in precancers of the breast.
Proteomic analysis of breast tissue and nipple aspirate fluid for breast cancer detection.
Proteomics of nipple aspirate fluid, breast cyst fluid, milk, and colostrum.
Black cohosh does not exert an estrogenic effect on the breast.

**2008** Nipple aspirate fluids from women with breast cancer contain increased levels of group IIa secretory phospholipase A2.
Celecoxib concentration predicts decrease in prostaglandin E2 concentrations in nipple aspirate fluid from high risk women.
Biomarkers associated with breast cancer are associated with obesity.
Increased shedding of soluble fragments of P-cadherin in nipple aspirate fluids from women with breast cancer.
uPA is upregulated by high dose celecoxib in women at increased risk of developing breast cancer.

**2009** Identification of a beta-casein-like peptide in breast nipple aspirate fluid that is associated with breast cancer.

**2010** Nipple aspirate fluid proteome of healthy females and patients with breast cancer.
Carbohydrate antigens in nipple aspirate fluid predict the presence of atypia and cancer in women requiring diagnostic breast biopsy.
Nipple aspirate fluid and ductoscopy to detect breast cancer.
Quantitative evaluation of DNA hypermethylation in malignant and benign breast tissue and fluids.

**2012** Breast cancer biomarkers predict weight loss after gastric bypass surgery.
Proteins and carbohydrates in nipple aspirate fluid predict the presence of atypia and cancer in women requiring diagnostic breast biopsy.

**2013** Vitamin D favorably alters the cancer promoting prostaglandin cascade.

**2016** Vitamin D3 Treatment Influences PGE2 and TGFI in Normal and Increased Breast Cancer Risk Women.

**2020s**

**2021** Using Organ Specific and Circulatory Biofluids to Screen Individuals at High Risk for Breast Cancer Presents Unique Challenges and Opportunities

**Figure S4**

## Timeline of Van Diest and Van der Wall

**2007** Oxytocin: bringing magic into nipple aspiration

**2008** Molecular analysis of nipple fluid for breast cancer screening

2010s

**2010** Successful oxytocin-assisted nipple aspiration in women at increased risk for breast cancer

**2015** Repeated nipple fluid aspiration: compliance and feasibility results from a prospective multicenter study

**2016** DNA promoter hypermethylation in nipple fluid: a potential tool for early breast cancer detection

**2019** Application of Nipple Aspirate Fluid miRNA Profiles for Early Breast Cancer Detection and Management

2020s

**2020** The Physiological MicroRNA Landscape in Nipple Aspirate Fluid: Differences and Similarities with Breast Tissue, Breast Milk, Plasma and Serum

**2021** Lessons Learned from Setting Up a Prospective, Longitudinal, Multicenter Study with Women at High Risk for Breast Cancer
The changing microRNA landscape by color and cloudiness: a cautionary tale for nipple aspirate fluid biomarker analysis
